# Characteristics of Legionnaires’ Disease Cases Hospitalized at a Specialized Infectious Disease Hospital, 2023–2024, with a Focus on Clusters Associated with Travel to a Spa Resort

**DOI:** 10.3390/microorganisms14040935

**Published:** 2026-04-21

**Authors:** Constanța-Angelica Vișan, Gina Filip, Carmen-Cristina Vasile, Anca Cristina Drăgănescu, Gheorghiță Jugulete, Anca Streinu-Cercel, Andreea Tudor, Laurențiu Mihăiță Stratan, Daniela Pițigoi, Ana Maria Tudor

**Affiliations:** 1 Faculty of Dentistry, Department of Infectious Diseases, “Carol Davila” University of Medicine and Pharmacy, 050474 Bucharest, Romania; angelica.visan@umfcd.ro (C.-A.V.); gheorghita.jugulete@umfcd.ro (G.J.); ana.tudor@umfcd.ro (A.M.T.); 2 National Institute for Infectious Diseases “Prof. Dr. Matei Bals”, 021105 Bucharest, Romania; carmen-cristina.vasile@drd.umfcd.ro (C.-C.V.); anca.draganescu@umfcd.ro (A.C.D.); anca.streinucercel@umfcd.ro (A.S.-C.); andreea-ioana.tudor@rez.umfcd.ro (A.T.); laurentiumstratan@gmail.com (L.M.S.); 3 National Institute for Mother and Child Health “Alessandrescu-Rusescu” (NIMCH “Alessandrescu-Rusescu”), 120 Lacul Tei Boulevard, 2nd District, 020395 Bucharest, Romania; filipginaadriana@yahoo.com; 4 Faculty of Medicine, Department of Epidemiology I, “Carol Davila” University of Medicine and Pharmacy, 021105 Bucharest, Romania; 5 Faculty of Medicine, Department of Pediatrics, “Carol Davila” University of Medicine and Pharmacy, 050474 Bucharest, Romania; 6 Faculty of Medicine, Department of Infectious Disease, “Carol Davila” University of Medicine and Pharmacy, 050474 Bucharest, Romania

**Keywords:** Legionnaires’ disease, cluster, travel-associated infectious diseases

## Abstract

Legionnaires’ disease is a rare cause of atypical pneumonia associated with a high mortality rate among untreated patients. In Romania, the disease has historically been underreported due to insufficient surveillance and limited diagnostic capacity. The aim of this study was to describe the characteristics of Legionnaires’ disease cases admitted to a specialized infectious disease hospital between 2023 and 2024, with a particular focus on a cluster associated with travel to a spa resort. Most cases included in our study (31/36) were confirmed by urinary antigen testing, while one case was confirmed by a significant increase in the level of specific antibodies against *Legionella pneumophila* serogroup 1 in paired serum samples. The most frequently reported symptom was fever (28/32), followed by chills (24/32). Among the 32 confirmed cases, 3 patients died. Two cases were identified as part of a family cluster involving a father and son who had undergone physiotherapy at a balneary resort. Both patients presented with fever and gastrointestinal symptoms, and radiological investigations confirmed mixed pneumonia associated with an intense inflammatory syndrome. In the father’s case, hepatic involvement and interstitial nephritis were also identified. Early diagnosis based on epidemiological data, clinical predictive scores, and laboratory investigations would allow timely administration of targeted antibiotic therapy and may contribute to reduced mortality.

## 1. Introduction

Legionnaires’ disease continues to be a public health problem because of its high potential for transmission through the inhalation of contaminated particles from man-made water systems. Legionnaires’ disease can affect a large number of vulnerable individuals and is associated with high mortality in untreated cases. The diagnosis can be difficult due to the nonspecific clinical manifestation and the lack of adequate tests, with Legionellosis being widely regarded as both underdiagnosed and underreported, throughout the entire European region.

*L. pneumophila* is an environmental Gram-negative bacterium that persists within biofilms and multiplies inside free-living protozoa. When aerosols contaminated with bacteria are inhaled, this opportunistic pathogen infects alveolar macrophages in the lungs, leading to severe pneumonia [1].

*L. pneumophila* was first identified following an outbreak of pneumonia in 1976 during an annual convention of the American Legion in Pennsylvania, United States. During this outbreak, an unknown respiratory disease affected 221 participants, resulting in 34 deaths. Since the identification of this pathogen, many more serogroups have been described [2].

*L. pneumophila* serogroup 1 is responsible for 80–90% of the cases reported in Europe and the United States. *L. longbeachae* accounted for approximately 1% of the cases worldwide at the beginning of 2000 but was on the rise in Europe, accounting for 50–60% of the cases in Australia and New Zealand [3].

The characteristics of *L. pneumophila* bacteria include multiplication in stagnant water, intrinsic resistance to biocides, biofilm formation, a symbiotic relationship with protozoa, and intracellular multiplication. Clinical manifestations can include infection of other organs in addition to the respiratory tract, such as the heart, nervous system, liver and soft tissues [4].

In 2023, a total of 14,537 cases of Legionnaires’ disease, with an incidence rate of 3.2 per 100,000 people (the highest rate ever), were reported in 30 countries from the European Union and European Economic Area. Four states (France, Germany, Italy and Spain) accounted for 72% of all reported cases [5].

The majority of cases (77%) reported in 2021 were community-acquired, 10% were travel-associated, 5% were nosocomial cases, 5% were of unknown origin and 3% were reported as “other”.

Eight countries (Belgium, Germany, Italy, Finland, France, the Netherlands, Portugal and Spain) reported at least one outbreak in 2021, with 3–18 cases each (137 cases in total). More than one-third of the outbreaks were community-acquired, which is a marked increase compared with previous years [6].

The mortality rate in European countries reported in 2021 was 9% [7]. At the global level, the reported mortality rate varies between 4% and 14% but can reach as high as 75% in vulnerable populations and specific contexts [8].

Data from Romania are scarce, mainly because of insufficient surveillance and diagnostic capacity. According to the National Centre for Surveillance and Control of Communicable Diseases (NCSCCD) reports, during 2023–2024, 113 cases of pneumonia with *L pneumophila* were reported in Romania, with an incidence of 0.3%000 inhabitants [9,10]. Most of the reported cases occurred sporadically and were locally acquired. A single outbreak was reported with two cases from the same family and seven imported cases (two cases had exposure in Italy and three cases in the United Arab Emirates, one each in Turkey and Albania).

Only one case was reported in a child, and the rest were adults.

However, infection with *L. pneumophila* has rarely been proven in children, representing less than 0.02% of every 100,000 individuals in most countries, including Romania. For example, the incidence of legionellosis in children reported in European countries was less than 0.25% for 100,000 individuals in 2021 [7,11].

The most recent medical literature highlights the risk of underestimation of legionellosis, as many countries do not have adequate diagnostic methods. The detection of *Legionella* spp. antigen in urine samples constitutes the primary diagnostic technique used in our country. In addition, the isolation of *Legionella* spp. requires the use of selective media that inhibit the growth of normal flora [12]. Many authors showed that real-time PCR is more sensitive than culture or urine antigen testing for identifying different *Legionella* strains in respiratory samples and wastewater systems [13,14].

The aim of this study is to present the characteristics of Legionnaires’ disease cases admitted to a specialized infectious disease hospital for two years, between 2023 and 2024, and to describe a cluster of two cases, a father and son, associated with travel to a spa resort. Also, we aimed to highlight the heterogeneity of clinical symptoms across different age groups and to increase awareness of the underdiagnosis of this disease among physicians from specialties other than infectious diseases, particularly pediatricians.

## 2. Materials and Methods

### 2.1. Study Design

A retrospective descriptive study was conducted using epidemiological surveillance data and medical records of patients who were diagnosed with legionellosis and admitted to the largest infectious disease hospital in Romania [15] between January 2023 and December 2024. In addition, two epidemiologically linked—cases of *L. pneumophila* pneumonia that occurred in a father and his son following exposure at a balneotherapy resort were analyzed in detail.

### 2.2. Data Gathering and Case Definitions

In Romania, healthcare professionals are legally required to immediately report all suspected and confirmed cases of legionellosis to district public health authorities, to facilitate epidemiological investigation and the implementation of prevention and control measures [16]. A single reported case represents the threshold for an epidemiological alert and triggers immediate public health intervention. Public health authorities subsequently perform a risk assessment and notify accommodation facilities potentially associated with the reported exposure.

Cases of legionellosis are classified at the local public health department level as probable or confirmed according to the national surveillance methodology and the European Union case definitions for legionellosis [17].

A confirmed case represents any person presenting with pneumonia and at least one of the following laboratory criteria for case confirmation: isolation of *Legionella* spp. from respiratory secretions or any normally sterile site, detection of *L.pneumophila* antigen in urine or a significant rise in specific antibodies against *L.pneumophila* serogroup 1 in paired serum samples.

A probable case represents any person presenting with pneumonia and at least one of the following laboratory criteria for a probable case: detection of *L.pneumophila* antigen in respiratory secretions or lung tissue, for example, by direct fluorescent antigen staining using monoclonal antibody-derived reagents; detection of *Legionella* spp. nucleic acid in respiratory secretions, lung tissue or any normally sterile site; a significant increase in specific antibody levels to *L. pneumophila* other than serogroup 1 or other *Legionella* spp. in paired serum samples. A single high level of specific antibody against *L. pneumophila* serogroup 1 in serum was also considered.

The national surveillance methodology defines a cluster of Legionnaires’ disease cases in accordance with the European Legionnaires’ Disease Surveillance Network as two or more cases of travel-associated Legionnaires’ disease in which the patients stayed at or visited the same commercial accommodation site within a 2–10-day incubation period before illness onset, with onset dates occurring within the same 2-year period [18].

According to national legislation, all suspected and confirmed cases must be immediately reported to public health authorities and subsequently entered into the European Surveillance System (TESSy).

### 2.3. Diagnostic Tools for Case Presentation

The diagnosis of pneumonia was based on the presence of respiratory symptoms and positive findings on chest X-ray and/or CT. Cases were confirmed by a urinary antigen test for *L. pneumophila* serogroup 1 (Immuview, SSI Diagnostica A/S, Hillerod, Denmark) or by nucleic acid amplification tests (Allplex Respiratory Panel, Seegene, Seoul, Republic of Korea).

To assess severity, we used the A-Drop score, in which six clinical features are assessed: age, dehydration, respiration, orientation and systolic blood pressure, assigning a score of 1 if abnormal. The sum of the scores has the highest value of six. The cutoff for severe outcomes was at least three points [19]. In accordance with [1], to assess the risk of *L. pneumophyla* pneumonia we used the Japanese Respiratory Society (JRS) score. JRS guidelines for the management of pneumonia, the *Legionella* pneumonia suspicion score comprises 6 items: 1. Male, 2. No coughing; 3. Difficulty breathing, 4. CRP ≥ 18 mg/dL; 5. Na value ≤ 134 mmol/L, 6. LDH value ≥ 260 U/L. Each item is assigned a score of 1 point. When the score is 3 or higher, *Legionella* pneumonia is suspected [20].

### 2.4. Statistical Analysis

The descriptive analysis was performed using Microsoft Excel. The continuous variables were presented using interquartile range or range values and categorical variables using frequencies.

#### Ethical Considerations

The study protocol was approved by the Institutional Bioethics Committee (approval number C14682/19 December 2025). Written informed consent was obtained from all participants or their legal guardians. Access to patient data was restricted to the infection prevention and control team and treating clinicians to ensure confidentiality.

## 3. Results

**Figure 1:**
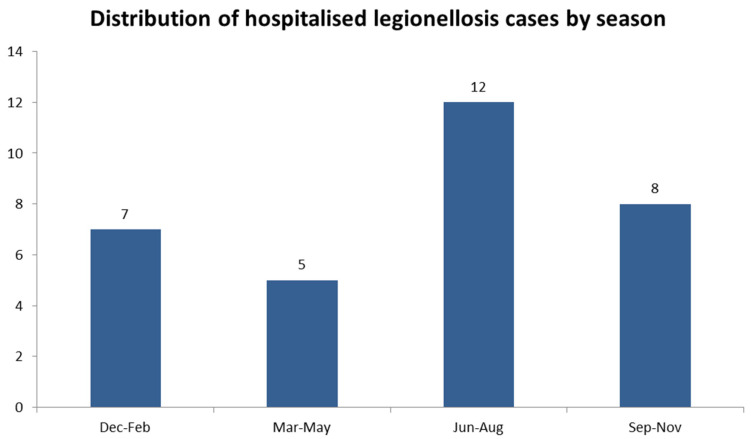
Distribution of hospitalized legionellosis cases by season during 2023–2024 (n = 32).

A total of 36 patients who were diagnosed with legionellosis were hospitalized at the NIID for two years, between 2023 and 2024, and were reported to the local health department. In most cases, 31 out of 36 (86.11%), were confirmed by urinary antigen testing; 1 case was confirmed by a significant increase in the specific antibody level to *L.pneumophila* serogroup 1 in paired serum samples, whereas 4 cases were classified as probable on the basis of laboratory criteria and were detected as *Legionella* spp. nucleic acid in respiratory secretions. The characteristics of the 32 confirmed cases are presented below in Figure 1.

A total of 17 cases were reported in 2023, and 15 were reported in 2024. In both analyzed years the highest number of cases was recorded in June (six cases in total), followed by August and October, with five cases. More than one-third of the cases (12/32) were reported during the summer (June–August), followed by eight cases during autumn (September–November), seven cases during winter (December–February) and five cases during the spring (March–May) (Figure 1).

The ages of the patients ranged from 5 to 87 years, with a median age of 60 years (IQR = 18.5). The cases were reported to the public health departments of six counties on the basis of the home addresses of the infected individuals, almost half of them, 15 out of 32, were living in Bucharest. The characteristics of hospitalized legionellosis patients are presented in Table 1, showing a predominance of male patients, 20 out of 32, older than 50 years, 25 out of 32 and living in the capital city, 15 out of 32.

The most common symptom reported was fever (28/32), followed by chills (24/32). Dyspnea and cough were presented in 20/32 patients. Four patients presented with chest pain. Hepatic involvement was reported in three patients (elevated liver enzymes, jaundice, and coagulation abnormalities). Gastrointestinal symptoms (diarrhea, abdominal pain and vomiting) were reported in five patients, whereas renal involvement (oliguria and hematuria) was present in two patients. One case presented with a rash. Among the 32 patients with confirmed legionellosis, 3 died.

Possible exposure based on patient declaration was identified in 18/32 cases. The most common was exposure to air conditioning, either at home or at the workplace (8/32) followed by occupational exposure (plumbing, 4/32), travel associated (5/32) and exposure to natural sources of water (fishing, 1/32). Four cases were associated with travel to spa resorts in Romania. One case was associated with travel to Dubai, United Arab Emirates and was classified as an imported Legionnaire’s disease case. Two of the cases were reported as part of a family cluster and are presented below. No nosocomial cases were reported.

### 3.1. Case Description

#### 3.1.1. Patient 1

A 51-year-old patient was examined in the Emergency Department of Tertiary Hospital in Bucharest on the fourth day of illness for headache, nausea and abdominal pain. Investigations performed showed leukocytosis with neutrophilia, negative rapid SARS-CoV-2 antigenic test. The abdominal sonography revealed hepatic steatosis, lipomatous infiltration of the pancreas, and otherwise normal cardiothoracic X-rays. He received saline solution (0.9% NaCl) via intravenous perfusion for dehydration, and he was discharged with the recommendation of symptomatic treatment.

The patient continued to experience chills, dizziness, pulsatile frontal headache, periumbilical colic and associated dry cough, myalgia, fatigability, vomiting, and loose stools; thus, he was admitted to our hospital on the sixth day of the disease.

His medical history noted paroxysmal supraventricular tachycardia corrected with ablation more than eight years ago, which was stable with 50 mg of metoprolol daily.

The patient stated that he spent 3 days with his family at a spa resort where he was exposed to aerosols from a thermal bath and the sauna. The first symptoms (fever and gastrointestinal symptoms) appeared 3 days after the patient returned from the resort. At the time of presentation at the hospital, none of the family members (wife and 5-year-old son) showed any signs of illness.

Clinical examination at admission revealed an unwell patient with a low-grade fever of 37.3° Celsius, overweight (body weight = 96 kg, height = 1.78 m, body mass index = 30.29 kg/m^2^), normal mental status, paleness, no cyanosis, a normal respiratory rate of 21/min, a normal peripheral oxygen level of 97% in ambient air, no dyspnea, fine rales at the basal right hemithorax, normal clinical cardiac auscultation, and normal arterial pressure, with no significant variation following position changes (standing at 120/75 mmHg and laying down at 130/80 mmHg, pulse at 80–85/minute), normal diuresis, and no meningeal signs. Neurologic consultation revealed no abnormal findings.

Investigations performed at admission revealed leukocytosis, neutrophilia, a high neutrophil/lymphocyte ratio, markedly elevated inflammatory markers, hepatic cytolysis, and hematuria, suggesting sepsis (Table A1).

**Figure 2:**
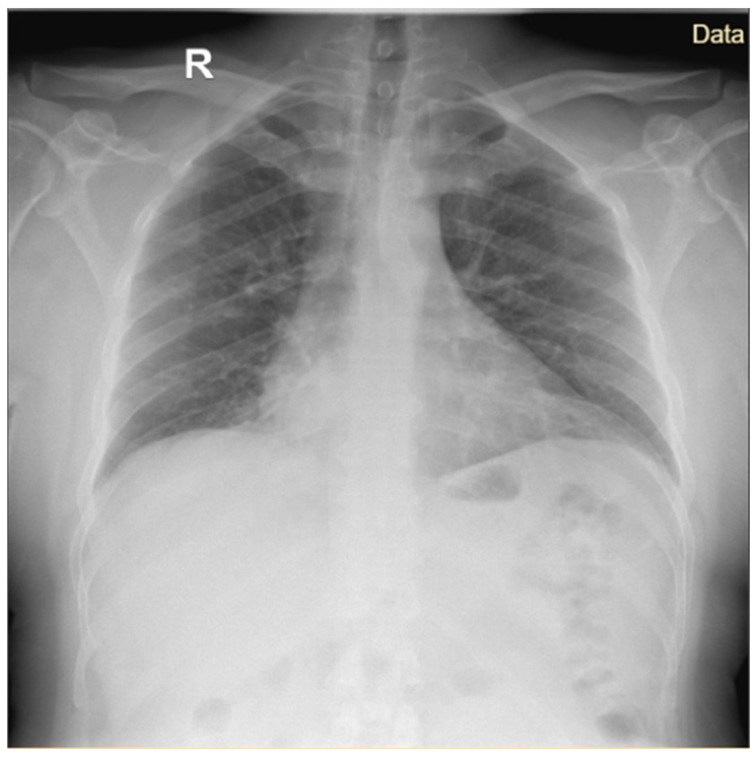
Anteroposterior cardiothoracic radiography Case 1.

An X-ray performed at admission revealed a reticulonodular pattern associated with diffuse alveolar opacities, predominantly on the right side rather than on the left side (Figure 2).

Considering the patient’s signs and symptoms and the epidemiological link, exposure to aerosols at a commercial balneotherapy accommodation site, 7 days before illness onset, the clinical diagnosis was acute pulmonary legionellosis complicated with nephritis and hepatitis and he received a course of ampicillin sulbactam IV plus oral doxycycline.

Microbiologic tests revealed positive results for *L. pneumophila* serogroup 1 urinary antigen. The A-Drop score in the adult patients suggested moderate disease (Table 2).

The clinical outcome was good; he was afebrile after 24 h and most of the symptoms ameliorated, except for the cough. The follow-up laboratory tests are included in Table A1 and revealed improved inflammatory syndrome and imaging findings.

The patient was discharged after eight days of hospitalization, with the recommendation to continue antibiotic treatment with oral ampicillin sulbactam (1.5 g) every 8 h plus doxycycline (100 mg) every 12 h for seven more days. At the follow-up visit, the patient had completely recovered.

#### 3.1.2. Case 2

Two weeks later, on the 27th of June, a 5-year-old male child was admitted to our clinic for symptoms that started two weeks earlier, with a high fever of up to 40 °C and abdominal pain. Prior to admission, the child had been evaluated in an emergency department at a pediatric tertiary hospital and was discharged with antibiotic treatment (cefixime) and antipyretics. After one week, the symptoms persisted; at the second evaluation, the antibiotic therapy was switched to clarithromycin. The fever persisted with the addition of chills and profuse night sweats after 36 h—during which he received three doses of clarithromycin—and he was hospitalized.

The medical history noted chickenpox two months before admission. No other chronic conditions were mentioned, except for histamine intolerance.

On physical examination at the time of admission, the child appeared acutely ill, well nourished (body weight of 19 kg), and febrile (39 °C), with pale skin with no rashes and a rare dry cough. His heart sounds were rhythmic and regular. Pulmonary auscultation revealed that the vesicular murmur presented bilaterally without additional crackles, a congestive pharynx, normal bowel transit, soft and mobile abdominal respiration, normal diuresis, consciousness, cooperation, and no meningeal signs.

It was further noted that the child’s father was hospitalized in our clinic two weeks earlier with a diagnosis of legionellosis (Legionnaires’ disease).

**Figure 3:**
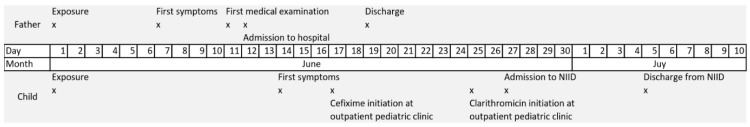
Timeline of clinical cases.

The child was exposed to aerosols at the spa approximately 14 days prior to the first symptoms (high fever and abdominal pain) and three weeks prior to admission (Figure 3).

Laboratory tests at admission disclosed increases in the following inflammatory markers: leukocytosis (11.500/mm^3^), neutrophilia (6.900/mm^3^), monocytosis (900/mm^3^), C reactive protein (53.6 mg/L), procalcitonin (0.73 ng/mL), erythrocyte sedimentation rate (46 mm/h), fibrinogen (405.2 mg/dL), mild hepatic cytolysis (ALT at twice the upper limit of normal), and lactic dehydrogenase (302 U/L).

Considering the similar spa aerosol exposure and father’s diagnosis, the child underwent serologic testing, and the results revealed positive IgM antibody for *L. pneumophila* and a negative urinary antigen test.

Serologic tests for *Mycoplasma pneumoniae* and *Chlamydia pneumoniae* were negative.

Other possible viral etiologies of pneumonia were excluded by the use of a multiplex respiratory virus panel, which was negative.

Two blood cultures were collected during the febrile episode prior to antibiotic administration, as were nasal and pharyngeal exudates, both of which were negative.

**Figure 4:**
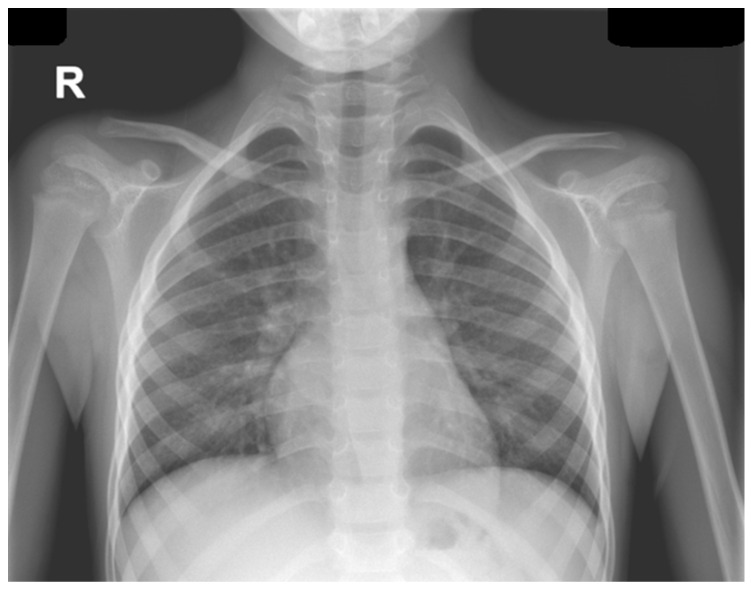
Anteroposterior cardiothoracic radiography, Case 2.

Chest radiography visualized the bilateral enlarged hila, predominantly on the right, with hilar and latero-basal alveolar and interstitial infiltrates, and abdominal ultrasound examination revealed mild hepatosplenomegaly, aerocolia, biliary sludge, and a 10 mm fluid collection in the Douglas pouch and an accessory spleen. Echocardiography excluded possible cardiac malformation, pericardial effusion or any evidence of pulmonary hypertension (Figure 4).

Given that the patient had been treated with a third-generation cephalosporin for 7 days prior to admission and that biliary sludge was present, empiric antibiotic therapy with meropenem associated with clarithromycin was initiated. After 24 h, the fever began to decrease. Following positive *Legionella* spp. serology results, clarithromycin therapy was continued for up to 10 days. The outcome was clinically and biologically favorable. A follow-up evaluation at 7 days after discharge revealed normal clinical and biological parameters. The child had A-DROP scores corresponding to a mild form of pneumonia (Table 2).

## 4. Discussion

Legionnaires’ disease remains an underdiagnosed and underreported infectious disease in Romania despite mandatory notification requirements. According to national legislation, all suspected and confirmed cases must be immediately reported to public health authorities and subsequently entered into the European Surveillance System (TESSy). Nevertheless, the incidence of Legionnaires’ diseases reported in Romania (which was substantially lower than the European average (0.3 versus 3.2 cases per 100,000 inhabitants), suggests an important number of underdiagnosed cases.

The increasing development of tourism infrastructure, including spa resorts and balneotherapy facilities, as well as the widespread use of air-conditioning and cooling systems, may contribute to favorable environmental conditions for *Legionella* spp. proliferation. These factors increase the risk of sporadic cases and clusters associated with travel or recreational activities. The number of Legionnaires’ disease cases correctly diagnosed and reported remains low because there is no diagnostic capacity at the level of all hospitals or public health laboratories [18].

Our retrospective analysis revealed a relatively stable number of hospitalized cases in our institute during the two consecutive years of analysis (17 cases in 2023 and 15 cases in 2024). These findings are consistent with national surveillance data reporting 56 cases in 2023 and 57 cases in 2024. However, the national epidemiological trend between 2014 and 2024 shows considerable variation, with peaks reported in 2018 and 2024, while fewer than 20 cases have been reported in several other years [9,10].

The age distribution observed in our study is consistent with international epidemiological data. Most patients (78.1%) were older than 50 years, reflecting the known increased susceptibility among older adults and individuals with underlying comorbidities. Similarly, European surveillance data have indicated that more than 90% of reported cases occur in individuals older than 45 years [5]. Comparable results were also published in the national annual surveillance reports for Legionnaire’s disease, with a median of 57 for both 2023 and 2024 [9,10]. Legionellosis remains rare in children and often leads to delayed diagnosis in pediatric populations. The pediatric case described in our study was the only pediatric case reported nationally during the two-year study period, highlighting the rarity of this infection in children [21].

The male/female ratio in our study (1.66) was similar to the national data (1.1), while the ECDC annual epidemiological report showed a higher male/female ratio (range 2.3–2.4:1, from 2017 to 2021) [5,9,10].

The peak number of cases found in our study occurred in June and August, partially overlapping with t the national data showing peaks in April, August and December [9,10] or with the observed seasonality at the EU level, with the highest number of confirmed Legionnaires’ diseases (61%) reported between June and October 2021.

Information regarding possible risk exposures in the 2–10 days prior to onset was obtained for 66.3% (75 cases) of the confirmed cases (113) at the national level in 2023–2024. The majority of cases (31/75%) reported living or working in buildings with air conditioning systems, 14 cases (18.7%) had visited swimming pools or spa areas, 11 cases (14.7%) had been in contact with natural water sources (lakes, ponds). Ten cases (13.4%) had occupational exposure [9,10]. The observed seasonality could be correlated both in our study and at the national level with the most common exposure declared by patients, as air conditioning exposure and exposure to natural water sources are more prevalent during the summer months.

Regarding the presented cases, initially, a potential connection between the father’s recent infection and the child’s symptomatology was not considered. For this reason, the patient received a broad-spectrum beta-lactam antibiotic as the first-line therapy.

On the other hand, the scientific literature mentioned Legionnaires’ disease as a rare cause of pneumonia in children; thus, pediatricians rarely include it in the differential diagnosis of lower tract infections. An important clinical clue for atypical bacterial infection was the persistence of fever and respiratory signs during antibiotherapy with beta-lactams [21].

Extrapulmonary manifestations, gastrointestinal, myalgia and confusion were present in both presented cases.

The diagnostic approach for the adult patient revealed that the following clinical signs were suggestive of atypical bacterial or viral pneumonia: gradual onset, dry cough, headache, and low-grade fever. Patients aged younger than 65 years, and digestive symptoms support atypical bacterial infection [22].

In the pediatric case, the pulmonary manifestations were subtle: a rare dry cough with normal pulmonary auscultation and a hyperemic pharynx. The diagnosis of pneumonia was confirmed by imaging investigations.

With respect to the biological investigation, while inflammatory tests revealed more important changes in the adult patient, despite the low-grade fever associated with hepatic and renal involvement, in the child patient, the inflammatory tests revealed less increased levels in the context of a very high fever (Table A1) [23,24].

In both presented cases, the JRS score for management of pneumonia suggested *L. pneumophila* infection, namely male gender, CRP higher than 18 mg/dL and LDH levels higher than 260 u/L; however, other etiologies could not be excluded, so broad-spectrum antibiotics associated with doxycycline or macrolide were given [21].

In the adult patient, the etiology was proven by the rapid *Legionella* spp. antigen test from the first day, whereas in the child patient, the rapid test was negative, and a positive result of the serologic test was available on the fourth day after admission, which confirms the usefulness of using combined methods to identify *L. pneumophila*. The use of PCR tests to identify the etiology would be beneficial for increasing the accuracy of the diagnosis [14].

Abdominal pain in the child may have been present from the onset as a manifestation of the disease, but its persistence may be explained by the presence of biliary sludge, which frequently occurs following treatment with cephalosporin, and by the presence of fluid in the rectovesical pouch. In the adult patient, an abdominal ultrasound did not reveal any abnormal findings; therefore, we considered this case to be an extrapulmonary manifestation of the disease.

The presence of the two cases of Legionnaires’ disease in the same family could have suggested the possibility of human-to-human transmission of the infection. Although *L. pneumophila* infection is transmitted through contaminated aerosols, there was a case of human-to-human transmission in the same family, described in an article published in 2016 [25].

However, we do not have enough data to support this hypothesis. The presence of both family members in the same environment, represented by a warm water system, makes the transmission of infection through aerosols and water droplets much more likely [25].

The specific aspects of the cases included a family cluster affecting a five-year-old boy, extrapulmonary manifestations, the need for hospitalization despite the initial appropriate antibiotic choice for the child, and the lag between admissions to the hospital, raising the suspicion of inter human transmission.

Our study provides information about the clinical and epidemiological characteristics of patients, the difficulty of diagnosis, and the necessity of quickly initiating specific antibiotic therapy. Importantly, doctors from all specialties should suspect Legionnaires’ disease in the presence of extrapulmonary manifestations and a history of aerosol exposure.

The strengths of our study were the ability to share data on a topic with little data from Romania and the presentation of clinical and epidemiological aspects regarding Legionnaires’ disease in hospitalized patients, including a child, rarely described in the literature. Also, we highlighted the heterogeneity of clinical symptoms in different age groups, in order to increase awareness of the underdiagnosis of this disease. Moreover, the patients included in the study represented 28% of the total national reported cases because of laboratory capabilities to diagnose legionellosis. Presented cases brought attention to an undiagnosed disease, especially in the pediatric population.

The limitations of our study are related to its retrospective design, which resulted in a high rate of missing epidemiologic linkage data. Additionally, the inclusion of only 31 hospitalized patients from a single hospital with trained personnel in infectious diseases and a high capacity microbiology laboratory limited the generalizability of our findings to the national surveillance network.

## 5. Conclusions

The family cluster described in this study illustrates the potential role of spa and balneotherapy facilities as sources of aerosolized *Legionella* spp. Such environments are recognized risk settings because warm water systems and aerosol-generating devices can facilitate bacterial proliferation and transmission. Early recognition of legionellosis based on epidemiological exposure, clinical presentation, and rapid diagnostic testing is crucial for initiating appropriate antimicrobial therapy. Prompt administration of targeted treatment has been shown to significantly reduce mortality, especially in severe cases. Improving diagnostic capacity, increasing clinician awareness, and strengthening environmental surveillance of water systems remain essential measures for improving the detection and prevention of Legionnaires’ disease in Romania.

## Figures and Tables

**Table 1:** Demographic characteristics of patients with confirmed legionellosis, between 2023 and 2024 (n = 32).

Characteristic	Number of Cases
Male	20/32
Female	12/32
**Age Group (years)**	
1–9	1/32
10–29	0/32
30–39	4/32
40–49	2/32
50–59	8/32
60–69	9/32
>70	8/32
**Residence**	
Bucharest (capital city)	15/32
Other districts	17/32

**Table 2:** A-DROP severity score in the studied patients at admission.

	Variable	Cut Offs	Adult Scoring	Child Scoring
A	Age	Male- over 65, Female over 75	0	0
D	Dehydration	BUN over 21 mg/dL	1	0
R	Respiration	SO_2_ 90% or less, PaO_2_ 60 or less	0	0
O	Orientation	Affected consciousness	0	0
P	Systolic Blood Pressure	BP 90 mmHg or less	0	0

## Data Availability

The original contributions presented in this study are included in the article. Further inquiries can be directed to the corresponding author.

## Appendix A

**Table A1:** Laboratory Test Results for Case 1 and 2 Described.

Test	Normal Value Reference	Adult Patient Results Day 1	Adult Patient Results Day 4	Adult Patient Results Day 8	Child Patient Results Day 1	Child Patient Results Day 5	Child Patient Results Day 8
Seric glucose	74–106 mg/dL	99	-	100	87	-	82
Blood urea nitrogen	19.26–42.8 mg/dL	52	-	33	16	26	30
Seric creatinine	0.66–1.25 mg/dL	0.8	-	1.0	0.3	0.4	0.3
Total Serum Protein	6.3–8.2 g/dL	7.1	-	-	7.3	-	-
Sodium serum	137–145 mmol/L	139	-	-	137	-	-
Potassium serum	3.6–5.0 mmol/L	4.6	-	-	4.0	-	-
CKMB	1–16 U/L	<3	-	-	-	-	-
AST/TGO	17–59 U/L	105	-	79	61	46	40
ALT/TGP	4–50 U/L	94	-	219	46	46	45
LDH	120–246 U/L	272	-	-	302	-	-
CK	55–170 U/L	132	-	-	82	-	-
GGT	15–73 U/L	108	-	133	-	-	-
Serum Lipase	23–300 U/L	63	-	-	-	-	-
Serum albumin	3.5–5 g/dL	3.8	-	-	4.1	-	-
Urine Erythrocytes	1–17/µL	206	-	-	-	-	-
Urine Leucocytes	1–40/µL	5	-	-	-	-	-
Urine Mucus	0–4/µL	212	-	-	6	-	-
Urine Epithelial Cells	1–28/µL	3	-	-	<1	-	-
Urine Bacteria	Absent	relative frequent	-	-	-	-	-
Urine Ketones	Negative	Trace	-	-	Negative	-	-
Urine Nitrites	Negative	Negative			Negative	-	-
Urine Urobilinogen	Normal	Normal			Normal	-	-
Urine biliary	Negative	Negative			Negative	-	-
Urine Glucose	Normal	Normal			Normal	-	-
Urine Protein	Normal	Small			Normal	-	-
Urine Density	1.015–1.030	1.025	-	-	1.010	-	-
Urine pH	5.5–6.5	5.5	-	-	5.5	-	-
Urine Ascorbic Acid	Negative	Negative	-	-	Negative	-	-
C-reactive protein	0–3.00 mg/L	178	204	27	53.6	23.3	17.3
White Blood Cells	3.6–9.6 × 10^3^/µL	14.23	11.04	7.84	11.5	12.1	11.9
Neutrophils Count	1.4–6.5 × 10^3^/µL	11.6	7.56	4.66	6.9	6.4	5.1
Lymphocytes Count	1.2–3.4 × 10^3^/µL	1.57	2.23	2.56	3.7	4.8	5.3
Neutrophil/lymphocyte count rate	<3	7.42	3.39	1.82	1.82	1.33	0.96
Monocytes Count	0–0.7 × 10^3^/µL	0.72	0.85	0.35	0.9	0.9	1.3
Eosinophils Count	0–0.7 × 10^3^/µL	0.13	0.14	0.15	0.0	0.0	0.2
Basophils Count	0–0.2 × 10^3^/µL	0.01	0.03	0.02	0.1	0.1	0.1
Red Blood Cells	3.9–5.7 × 10^3^/µL	4.81	4.66	4.94	4.39	4.60	4.46
Hemoglobin	12.1–17.2 g/dL	13.5	13.1	13.7	10.4	10.9	10.6
HCT	36.1–50.3%	41.1	39.8	42.5	31.6	32.8	31.8
MCV	82–97.4 fL	85.6	85.3	86.2	71.9	71.3	71.4
MCH	27.6–33.3 pg	28.2	28.0	27.8	23.7	23.6	23.7
MCHC	33–34.8 g/dL	32.9	32.8	32.2	32.9	33.0	33.2
RDW	11.6–13.7%	13.8	14.1	13.9	16.4	16.4	16.6
Platelets	200–400 × 10^3^/µL	199,000	217,000	329,000	299,000	468,000	501,000
MPV	7.8–11 fl	7.6	8.1	7.5	6.5	6.2	6.0
HDW	0–99.9%	2.87	3.05	2.97	-	-	0.1
PDW	0–99.9%	58.8	57.2	60.4	16.4	15.8	15.7
PCT	0–0.99%	0.15	0.17	0.25	-	0.29	0.30
APTT (sec)	24–38 s	27.1	-	-	-	-	-
PT (sec)	10.5–13 s	11.4	11.4	11.0	-	-	-
PT (%)	80–115%	99	99	104	-	-	-
INR	0.90–1.15	0.96	0.96	0.93	-	-	-
FIBRINOGEN	200–393 mg/dL	963	919	535	405	328	-
Procalcitonin	0–0.5 pg	-	-	-	0.73	-	-
Urinary *Legionella* spp. antigen/*Streptococcus pneumoniae*	Negative	Urinary *Legionella* spp. Antigen—Positive Urinary Antigen *Streptococcus pneumoniae*- Negative	-	-	Negative	-	-
Blood Culture	Negative	Negative	-	-	Negative- 2 samples	-	-

The results marked in red are out of normal reference levels.

## References

[1] Personnic N., Striednig B., Hilbi H. (2018). Legionella Quorum Sensing and Its Role in Pathogen–host Interactions. Curr. Opin. Microbiol..

[2] García-Rodríguez F.J., Buchrieser C., Escoll P. (2023). Legionella and Mitochondria, an Intriguing Relationship. Int. Rev. Cell Mol. Biol..

[3] Whiley H., Bentham R. (2011). Legionella Longbeachae and Legionellosis. Emerg. Infect. Dis..

[4] Viasus D., Gaia V., Manzur-Barbur C., Carratalà J. (2022). Legionnaires’ Disease: Update on Diagnosis and Treatment. Infect. Dis. Ther..

[5] Surveillance Atlas of Infectious Diseases. https://atlas.ecdc.europa.eu/public/index.aspx?Dataset=27&HealthTopic=30.

[6] Prevalence of Legionella as a Waterborne Pathogen and Its Health Impacts in the Pan-European Region. https://www.who.int/europe/publications/i/item/9789289062572.

[7] European Centre for Disease Prevention and Control (2021). Legionnaires’ Disease.

[8] Cooley L.A., Pondo T., Francois Watkins L.K., Shah P., Schrag S. (2020). Active Bacterial Core Surveillance Program of the Emerging Infections Program Network Population-Based Assessment of Clinical Risk Factors for Legionnaires’ Disease. Clin. Infect. Dis..

[9] Institutul Național de Sănătate Publică (2023). https://insp.gov.ro/download/analiza-bolilor-transmisibile-aflate-in-supraveghere-raport-pentru-anul-2023/.

[10] Institutul Național de Sănătate Publică (2024). https://insp.gov.ro/download/analiza-bolilor-transmisibile-aflate-in-supraveghere-raport-pentru-anul-2024/.

[11] Shim J.Y. (2020). Current Perspectives on Atypical Pneumonia in Children. Clin. Exp. Pediatr..

[12] CDC National Center for Immunization and Respiratory Diseases (NCIRD). https://www.cdc.gov/ncird/index.html.

[13] Ricci M.L., Grottola A., Fregni Serpini G., Bella A., Rota M.C., Frascaro F., Pegoraro E., Meacci M., Fabio A., Vecchi E. (2018). Improvement of Legionnaires’ disease diagnosis using real-time PCR assay: A retrospective analysis, Italy, 2010 to 2015. Eurosurveillance.

[14] Redwitz J., Streich P., Zamfir M., Walser-Reichenbach S.M., Seidel M., Herr C.E.W., Heinze S., Quartucci C. (2024). Verification and Application of qPCR and Viability-qPCR for Legionella Monitoring in Evaporative Cooling Systems Complementing the Conventional Culture Method. Sci. Total Environ..

[15] Vasile C.-C., Gheorghe L.-A., Chivu C.-D., Anghel M.A.M., Mîinea Ș.E., Pițigoi D., Crăciun M.-D. (2024). Clostridioides Difficile Infections and Antibiotherapy: Results of Four Years of Observation in a Romanian Tertiary Hospital. Microorganisms.

[16] HOTARARE 657 18/05/2022—Portal Legislativ. https://legislatie.just.ro/Public/DetaliiDocument/255558.

[17] EUR-Lex Commission Implementing Decision (EU) 2018/945 of 22 June 2018 on the Communicable Diseases and Related Special Health Issues to Be Covered by Epidemiological Surveillance as Well as Relevant Case Definitions. https://eur-lex.europa.eu/.

[18] Institutul Național de Sănătate Publică. https://insp.gov.ro/centrul-national-de-supraveghere-si-control-al-bolilor-transmisibile-cnscbt/metodologii/.

[19] European Legionnaires’ Disease Surveillance Network (ELDSNet). https://www.ecdc.europa.eu/en/about-us/partnerships-and-networks/disease-and-laboratory-networks/eldsnet.

[20] Mukae H., Iwanaga N., Horita N., Komiya K., Maruyama T., Shindo Y., Imamura Y., Yatera K., Yamamoto Y., Yanagihara K. (2025). The JRS Guideline for the Management of Pneumonia in Adults 2024. Respir. Investig..

[21] Legionella.org Legionnaires’ Disease in Children. https://legionella.org/legionnaires-disease-in-children/.

[22] van der Poll T., Opal S.M. (2009). Pathogenesis, Treatment, and Prevention of Pneumococcal Pneumonia. Lancet.

[23] Orfanu A., Popescu C., Tilişcan C., Streinu-Cercel A., Aramă V., Aramă Ş.S. (2020). The Usefulness of Neutrophil/lymphocyte Count Ratio in the Diagnosis and Prognosis of Bacterial Sepsis—An Old Parameter with New Implications. Rev. Romana Med. Lab..

[24] Kline M.W., Orange J.S., Giardino A.P., Rathore M., Harris Z.L., Cabrera A. (2026). Rudolph’s Pediatrics.

[25] Correia A.M., Ferreira J.S., Borges V., Nunes A., Gomes B., Capucho R., Gonçalves J., Antunes D.M., Almeida S., Mendes A. (2016). Probable Person-to-Person Transmission of Legionnaires’ Disease. N. Engl. J. Med..

